# Socioeconomic Status and Other Characteristics in Childhood Leukemia

**Published:** 2013-01-22

**Authors:** H Hashemizadeh, H Boroumand, R Noori, M Darabian

**Affiliations:** 1 Department of Nursing, Quchan Branch, Islamic Azad University, Quchan, Iran.; 2 Dr. Sheikh Hospital, Mashhad University of Medical Sciences, Mashhad,Iran.; 3 Department of Midwifery, Quchan Branch, Islamic Azad University, Quchan, Iran.

**Keywords:** Child, Leukemia, Social Class

## Abstract

**Background:**

Leukemia is the most prevalent childhood cancer, and Acute Lymphoblastic Leukemia (ALL) constitutes 75% of all cases. Some epidemiological studies have shown a relationship between socioeconomic status (SES) and some childhood cancers. In the present study, an attempt was made to assess socioeconomical status in a case-control study.

**Materials and Methods:**

In 2010, a case-control study was conducted on 100 cases of acute lymphoblastic leukemia aged 1 to14 years in Department of Pediatric Oncology of Dr.Sheikh Hospital in Mashhad – Iran and matched age and sex with 400 healthy controls. Data was collected by interview using a questionnaire. Ninety five percent confidence intervals were used to measure the relationship between childhood Acute Lymphoblastic Leukemia and parental education, income status, father's job (Socioeconomic status), number of children, birth score and paternal smoking.

**Results:**

There was a significant difference in parental education level, income status, and number of children, birth score, father's job and paternal smoking between two groups. Regression analysis showed that the risk of childhood ALL associated with paternal smoking, and father's high risk job. Fifty percent cases and thirty five percent of control groups located in upper lower and lower middle class of socioeconomic status, respectively. There is a meaningful different between socioeconomic status in two groups. But the risk of childhood ALL did not associate with socioeconomic status.

**Conclusion:**

The results suggest that paternal smoking and father’s high risk job are related to risk of childhood leukemia. It should be considered for planning support.

## Introduction

In the US, the incidence of ALL is roughly 6000 new cases per year, or approximately 1 in 50,000. ALL accounts for approximately 70 percent of all childhood leukemia cases (ages 0 to 19 years), making it the most common type of childhood cancer. ALL is slightly more common in males than females (1).

For unexplained reasons, the incidence of ALL is substantially higher in white children than in black children, with a nearly threefold higher incidence from age 2 to 3 years in white children compared with black children (2). Dramatic improvements in survival have been achieved in children and adolescents with cancer. Between 1975 and 2002, childhood cancer mortality has decreased by more than 50%. For ALL, the 5-year survival rate has increased over the same time from 60% to 89% for children younger than 15 years and from 28% to 50% for adolescents aged 15 to 19 years (3).

Some epidemiological studies have found a relationship between socioeconomic status (SES) and some childhood cancers, such as lymphoma or leukemia. In the present study, an attempt was made to assess socio-economical class in a case-control study investigating childhood leukemia. Over the last years, the interest in assessing social inequalities and health has increased substantially. Socioeconomic characteristics are associated with morbidity and mortality discrepancies in many developed countries (4). 

The relationship between social inequalities and cancer has been well studied for adults but less extensively for childhood cancer. Childhood leukemia seems to be unique in this aspect, as earlier it was reported that childhood leukemia was more frequent among individuals of low socioeconomic status (SES). A comprehensive review on the association between SES and childhood leukemia was recently published and the authors have pointed out that this association is likely to vary according to time, place and study design (5).

There are more positive associations (i.e., higher incidence rates among high SES) in older studies and negative associations in newer ones. Overall, studies conducted in Europe have shown a positive association between childhood ALL and SES, while North-American studies reported negative ones. Registry-based and record-based studies generally presented positive associations, whereas interview-based case–control studies showed negative associations (5, 6). The aim of this study was to assess the relationship between socioeconomic status and childhood leukemia.

## Materials and Methods

A case-control study was conducted on 100 cases of acute lymphoblastic leukemia age 1 to 14 years in department of pediatric oncology of Dr.Sheikh Hospital in Mashhad – Iran and matched on age and sex to 400 healthy controls. The control group consisted of four hundred children between the ages of 1 to 14 years without any malignancy, hematologic or chronic disorders, who were seen in the outpatient clinics for mild infectious diseases. The diagnosis of ALL was confirmed by an oncologist. Data was collected by interview using a questionnaire. We measured socioeconomic status by kuppuswamy’s socioeconomic status scale. In this scale, parental education, income status and father's job were measured and classified in five classes (Upper, Upper middle, Lower middle, Upper Lower and Lower).

**Statistical analysis**

Data analyzed by chi-square test and regression analysis. 95% confidence intervals were used to measure the relation between childhood ALL and parental education, income status, father's job (Socioeconomic status), number of children, birth score and paternal smoking.

## Results

Out of 100 patients with ALL, 23 % (n=23) had uneducated fathers, 55% (n=55) had Primary and secondary school, 18% (n=18) had diploma and4% (n=4) had above diploma. While of 400 children in controls, 8% (n=32) had uneducated fathers, 43% (n=172) had Primary and secondary school and 28% (n=112) had a diploma and 21% (n=84) had above diploma. There was a significant difference in father's education level between two groups by chi-square test (P-value=0.01).

Out of 100 patients with ALL, 34% (n=34) had uneducated mothers, 45% (n=45) had Primary and secondary school, 21% (n=21) had a diploma and no one had above diploma. While of 400 children in controls, 7% (n=28) had Uneducated mothers, 50 % (n=200) had Primary and secondary school and 26 % (n=104) had diploma and 17 % (n=68) had above diploma. There was a significant difference in mothers’ education level between two groups by chi-square test (P-value=0.02).

Family income state in two groups was significantly different by chi – square test (P-value =0.01). Eighty one % (n=81) of cases in ALL group and 21 % (n=84) of case group have low incomes. Also 19 % (n=19) of cases in ALL group and 79% (n=316) of case group were in the intermediate and high income category.

Among case fathers, 40 % reported having smoke, compared with 5 % of control fathers. Forty percent of parents of children in the study were smokers, while this was 5 % for fathers in control group There was a significant difference in paternal smoking between two groups by chi-square test (P-value=0.02).

Number of children in two groups was significantly different by chi-square test (P-value=0). The number of children in 26 % of cases and 38 % of controls were three and one respectively.

Birth score in two groups was significantly different by chi-square test (P-value=0). Birth score of children in 30% of cases and 59% of control were one.

There was significant difference between father′s high risk jobs in two groups (P-value=0.02). 27% (n=27) of patients in case and 3 % (n=12) in control group had fathers with high risk jobs. The type of high risk job in most of the ALL group were 74 % (n=20) farming and 26% (n=7) painting.

Regression analysis showed the risk of childhood ALL associated with paternal smoking (P-value =0, OR=12.6, CI 95%, 7.4-21.5) and father's job (P-value=0, OR=11.9, CI 95%, 6.2-22.9).

Table II shows most of cases (50%) and control group (35%) located in upper lower and Lower middle class respectively. There was a significant difference between socioeconomic statuses in two groups (P-value=0.01).

**Table I:** Odd Ratio contributes to risk factor of ALL. Regression analysis showed the risk of childhood A.L.L associated with paternal smoking, and father's high risk job

**Variable**	**odd Ratio**	**CI 95%**	**Test**	
			^2^ χ	P
**paternal smoking**	12/6	7/4 - 21/5	0	117/6
**father's high risk job**	11/9	6/2 – 22/9	0	95/7

**Table II:** Socioeconomic status in case and control group. Most of cases and control groups located in upper lower and lower middle class of socioeconomic status, respectively.

**Class**	**case**	**control**
	%( n)	%( n)
**Upper**	5%(5)	10 %(40)
**Upper middle**	10%(10)	25 %(100)
**Lower middle**	23%(23)	35 %(140)
**Upper Lower**	50%(50)	24 %(96)
**Lower**	12%(12)	6 %(24)
**Total**	100%(100)	100% (400)

## Discussion

Overall, studies utilizing area-based socioeconomic measures have demonstrated an increased risk of ALL among people with high SES (7).

 In Yorkshire, England, an ecological study performed on 248 children (<15 years) revealed a marked reduction in incidence of ALL for the highest quintile of deprivation (risk ratio = 0.67, 95% CI 0.44–1.01) (8). 

On the other hand, the results of the studies of SES and childhood leukemia using individual-level assessment are controversial (9- 12).

High levels of family income and parental education have been consistently associated with lower risk of childhood leukemia while the association of paternal occupational class with childhood leukemia demonstrates a contrary association (13, 14). A case-control study was conducted in the United Kingdom did not show any difference in childhood ALL risk according to deprivation levels, whether using area- or individual-based measure of SES (father's occupation), at the time of birth or diagnosis (15).

On the basis of their findings, the authors suggest that SES in the United Kingdom does not have influence on the development of ALL in children and that the previous findings could be artificial (15).

The result of study in Brazil showed the lower risk of ALL among children living in low SES areas (16).Their finding of lower childhood ALL rates among females in areas of higher population density is not compatible with results which were reported by Muirhead in 1995 for 3 metropolitan areas of the United States (17). However, a geographical analysis of the childhood ALL cases from north-west England has demonstrated a monotonic relationship between incidence rates and population density, with a higher risk in more densely populated areas (18). 

A research in Yazd about Socioeconomic status and childhood Leukemia showed that there was a significant difference in parental education level (P-value=0, P-value=0.001), income status (P-value =0.001) and father's job (0.002) between two groups (case and control). The risk of childhood ALL was associated with paternal smoking (P-value =0.001, OR=2.6, CI 95%, 1.5-4.5), alcohol drinking (P-value=0.003, OR=3.33, CI 95%, 2.7-3.9) and addiction (P-value=0, OR=42.7, CI95%, 5.56-328.34) (19). 

The association between parental smoking and risk of childhood acute lymphoblastic leukemia (ALL) was investigated in an Australian population-based case-control study that included 388 cases and 868 controls aged <15 years, recruited from 2003 to 2006. Data was analyzed by logistic regression. Maternal smoking was not associated with risk of childhood ALL, but the odds ratio for paternal smoking of ≥15 cigarettes per day around the time of the child's conception was 1.35 (95% confidence interval: 0.98, 1.86). Study results suggest that heavier paternal smoking around the time of conception is a risk factor for childhood ALL (20). 

Most previous studies of paternal smoking before pregnancy have reported an increased risk of ALL (21, 22). A study reported an association only for paternal smoking (23), while another found that the association was considerably stronger if the mother smoked postnatally (24). 

In this study we reported an association only for paternal smoking at home. A case-control study of children of ages 10 years and below in Los Angeles County was conducted to investigate the causes of leukemia. The mothers and fathers of acute leukemia cases and their individually matched controls were interviewed regarding specific occupational and home exposures as well as other potential risk factors associated with leukemia. 

Risk related to fathers exposure to chlorinated solvents, employment in the transportation equipment-manufacturing industry, and parents exposure to household or garden pesticides and incense remains statistically significant after adjusting for the other significant findings (25).

A case-control study was performed with 193 children who reside in Mexico City and had been diagnosed for ALL. With the results obtained from this study, they concluded that, among the children of fathers exposed to a high level of carcinogenic substances at work, there seemed to be a greater risk of developing ALL. The only occupation that showed a statistically significant increased risk was insurance agent. Occupations that remained as risky occupations during the four periods were the following: insurance agent, farmer, machinery operator, mechanic, packer, and builder (26). Mirmohammadi assessed the relation between environmental factors (Hydrocarbon, Agriculturaltoxin, and insecticide) and leukemia/lymphoma in children, which was evaluated by the frequency of the parents’ hazardous occupations, and their smoking, drug addiction and alcoholism habits by a case-control study. In case group, fathers occupations were mostly farmers 50 (58.1%), followed by painter or exposed to hydrocarbons 14 (16.6%), but in the control group, farmers were 17 (19.7%), and painters or those exposed to hydrocarbons were 5 (5.8%). The frequency of fathers' various occupations was significantly different between cases and controls. In addition, smoking, drug addiction and alcoholism was significantly higher in cases fathers than controls fathers (27).

## Conclusion

Connections of SES measures to childhood leukemia are likely to vary with place and time. Validation studies are needed to estimate SES-related selection and participation in case–control studies. Because of different socioeconomic measures (such as income and education), individual-level and ecological-level measures may represent different risk factors, we advise researchers to report these measures separately rather than in summary indices of social class (5).

The frequency of leukemia in children whose fathers were painters or farmers is higher than normal children. So people with these occupations should pay more attention and should be protected against these risk factors. 
